# A Retrospective Study of Disparities in Mortality Due to Diabetes Mellitus Over a 22-Year Period in the United States

**DOI:** 10.7759/cureus.91137

**Published:** 2025-08-27

**Authors:** Urveesh Sharma, Asma Zahra, Sayali Kulkarni, Sai Prashanthi Varakala, Adiba Khan

**Affiliations:** 1 Department of Medicine, Maulana Azad Medical College, New Delhi, IND; 2 Department of Internal Medicine, Jinnah Postgraduate Medical Centre, Karachi, PAK; 3 Department of Internal Medicine, Georgian National University, Tbilisi, GEO; 4 Department of Internal Medicine, Osmania Medical College, Telangana, IND; 5 Department of Internal Medicine, School of Health Sciences, University of Georgia, Tbilisi, GEO

**Keywords:** cdc-wonder, diabetes mellitus, metropolitan, rural, urban

## Abstract

Background: Diabetes mellitus (DM) is a chronic endocrine disorder characterized by impaired insulin production or resistance to its action, resulting in elevated blood glucose levels. Urban-rural mortality rate of DM signifies the understanding of health disparities by analyzing differences in health and well-being of populations living in urban and rural areas.

Methodology: A retrospective comparative study was conducted by collecting data from the CDC WONDER (Centers for Disease Control and Prevention Wide-ranging Online Data for Epidemiologic Research) database on August 24, 2022. ICD-10 (International Classification of Diseases, 10th Revision) codes E10-E14 were used, and the data were categorized into rural and urban deaths based on the 2013 urbanization classification. The variables used to study these differences were age, gender, and race.

Results: The mortality due to DM was higher in rural areas compared to urban areas for all age groups, genders, and races. Significantly higher crude mortality rates per 100,000 population were observed in rural areas among individuals aged 75-84 years (177.17) and +85 years (347.37), compared with other age groups. In rural areas, males (33.91) had a significantly higher mortality rate than females (33.10). Mortality rates were also significantly higher in rural areas among Black or African American individuals (43.75) and American Indian or Alaska Native individuals (40.85), compared with other racial groups.

Conclusions: This study reveals the significant disparities in urban versus rural mortality among patients with DM over 22 years. A substantial increase was seen in all races of individuals in the United States. There was a significant rise in mortality trends for both rural and urban populations in the year 2020, and a huge difference was seen in the mortality rate of American Indians in both urban and rural areas.

## Introduction

Diabetes mellitus (DM) is a chronic, non-communicable endocrine disorder that is increasingly prevalent and presents serious clinical challenges worldwide. It is often associated with complicated metabolic changes in patients [1]. In 2021, there were an estimated 529 million cases of diabetes globally, with a 95% confidence interval ranging from 500 to 564 million. The age-standardized prevalence of diabetes was 6.1%, with estimates ranging from 5.8% to 6.5% [2]. In the United States, approximately 38.4 million people, or 11.6% of the population, had diabetes in 2021. That year, diabetes was the eighth leading cause of death, listed as the primary cause on 103,294 death certificates, and mentioned as a contributing factor in a total of 399,401 certificates [3,4].

Significant disparities exist in diabetes care between rural and non-rural populations in the United States, though it remains unclear whether residing in rural areas affects diabetes mortality differently. Limited data on trends in diabetes mortality in rural populations further complicates efforts to develop targeted interventions [5]. Studying these disparities is crucial as it reveals differences in healthcare access and socioeconomic factors that impact disease outcomes. Addressing these disparities could lead to better healthcare access in rural areas, targeted interventions, and improved health equity, ultimately reducing mortality and enhancing diabetes care.

Despite the higher prevalence of type 2 diabetes (16% more) and diabetes-related hospital mortality (20% higher) in rural areas compared to urban areas, as well as slower improvement in overall mortality rates from 1999 to 2016, no studies have specifically compared diabetes mortality between urban and rural populations [6]. Rural patients often face healthcare inequalities that may contribute to variations in diabetes-related mortality between rural and urban regions [7]. To address this, we analyzed trends in diabetes-related mortality over the past 22 years using the CDC WONDER (Centers for Disease Control and Prevention Wide-ranging Online Data for Epidemiologic Research) database, focusing on differences across urban and rural areas based on year, age, gender, race, and census region.

This study aimed to analyze long-term (1999-2020) disparities in diabetes-related mortality between urban and rural U.S. populations using the CDC WONDER database, stratified by age, gender, and race

## Materials and methods

A retrospective comparative study focusing on the disparities in DM-related mortality between urban and rural areas over 22 years was conducted in the United States from the year 1999 to 2020 using the CDC WONDER underlying cause of death database [8]. The total number of deaths was seen to be 1,674,724 in a population of 6,746,356,647, with the crude death rate per 100,000 being 24.8. Data were extracted on August 24, 2024. CDC WONDER contains deidentified, publicly available data. As this research qualifies as non-human participant research, no ethics committee approval was needed.

Data were collected on August 24, 2024. In this retrospective study, the authors extracted CDC WONDER data from 1999 to 2020 on DM-related mortality disparities in urban and rural areas of the United States. The study followed the Strengthening the Reporting of Observational Studies in Epidemiology (STROBE) reporting guidelines [9]. The mortality was grouped by age group (10-year age range), gender (men or women), and race in the year (1999-2020). Based on the International Statistical Classification of Diseases and Related Health Problems (ICD-10) codes E10 to E14. Underlying cause of death is DM in 1999-2020; Underlying Cause of Death by Bridged-Race Categories, ICD-10 code no. (E10-E14) Under DM, E10 (Insulin Dependent DM), E11 (Non-Insulin Dependent DM), E12 (Malnutrition-related DM), E13 Other Specified DM, with coma, E14 (Unspecified DM). Diabetes-related mortality was identified using the ICD-10 codes E10-E14 as the cause of death [10]. The population was divided into Urban/ Metropolitan (Large Central Metro, Large Fringe Metro, Medium Metro, and Small Metropolitan areas) and Rural/Non-metropolitan areas; Micropolitan (Nonmetro) and Noncore (non-metro) counties according to the 2013 U.S. Census classification, based on the Office of Management and Budget’s February 2013 delineation of metropolitan statistical areas and micropolitan statistical areas [7]. The National Diabetes Statistics Report provides essential data to guide diabetes prevention and control efforts across the United States [11]. In this study, crude, age-adjusted mortality rates (AAMRs) and total mortality rates are presented per 100,000 population. Beginning with the 1999 data year, the National Center for Health Statistics adopted the age distribution of the 2020 population of the United States. This study presents the percent change in crude mortality rates and AAMRs between two time points (1999 and 2020), and identifies trends in AAMRs between 1999 and 2020 using the Pearson correlation trend test and its *P*-value using yearly data points. Statistical significance was set at the *P *< 0.05 level.

After grouping, data were exported to Microsoft Excel and analyzed using R (R Core Team, 2023). Variables were presented as frequencies and rates, and crude mortality rates, as well as AAMRs, were calculated per 100,000 population. The Pearson correlation trend test was applied to examine temporal trends in mortality from 1999 to 2020, with statistical significance set at *P* < 0.05. All plots were generated using the ggplot2 package (Springer-Verlag, New York, 2016).

## Results

Aggregate data of total deaths from 1999 to 2020 were obtained for DM from the CDC WONDER database, and the absolute number of reported mortalities in urban and rural areas due to DM from 1999 to 2020, as per the 2013 Urbanization Classification, was noted. The total number of deaths due to DM in rural areas was 337,367, and in urban areas was 13,37,357 during the interval 1999-2020 (Table 1).

**Table 1:** Absolute number of reported mortalities in urban and rural areas due to diabetes mellitus from 1999 to 2020, according to the 2013 Urbanization Classification. Data are represented as *n* (%).

Type	*n* (%)
Urban (metropolitan area)	1,337,357 (79.9%)
Large central metropolitan	458,413 (34.3%)
Large fringe metropolitan	345,997 (25.9%)
Medium metropolitan	359,942 (26.9%)
Small metropolitan	173,005 (12.9%)
Rural (non-metropolitan area)	337,367 (19.1%)
Micropolitan	188,622 (55.9%)
Non-core	148,745 (44.1%)

Among the urban areas, mortality rates were highest in the large central metropolitan areas (34.3%), with the lowest rates observed in small metropolitan areas (19.1%). Among rural areas, the non-core areas had higher mortality rates (55.9%) than the micropolitan areas (44.1%).

**Figure 1:**
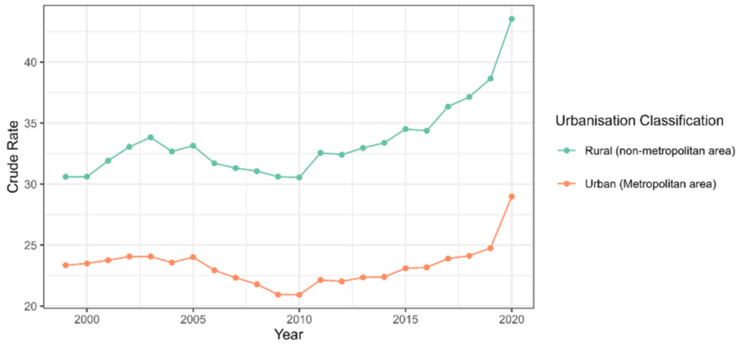
Line diagram showing trends in urban versus rural mortality due to diabetes mellitus calculated in crude rate per 100,000 population.

The line diagram in Figure 1 shows the trends in urban versus rural mortality due to DM calculated in crude rate per 100,000 population (Figure 1). The mortality in rural areas is consistently higher than in urban areas in all years. The mortality in rural areas seems to have been rising from 1999 to 2020, except the year 2010, when there was a slight drop in mortality. The mortality in urban areas seems to be rising from 1999-2020, except the year 2010, when there was observed a slight drop in mortality was observed.

The mortality due to DM was higher in rural areas compared to urban areas for all age groups, gender, and race (Table 2). Significantly higher mortality rates were seen in rural areas in the age group of 75-84 years (177.17) and 85+ years (347.37) compared to other age groups. Significantly higher mortality rate was seen in rural areas in males (33.91) compared to females (33.1). Significantly higher mortality rates were observed in rural areas among Black or African Americans (43.75) and American Indian or Alaska natives (40.85) compared to other races.

**Table 2:** Mortality due to diabetes mellitus in urban and rural areas based on age, gender, and race. *P*-value < 0.05 was considered statistically significant Mortality rates were calculated by dividing the number of deaths (*n*) in the population by the total population.

Variables	Urban			Rural			Binomial test
	Mortality	Total population	Mortality rate	Mortality	Total population	Mortality rate	*P*-value
Age groups							
<1 year	36	46,611,387	0.08	NA	NA	NA	NA
1-4 years	82	299,561,638	0.03	30	49,008,275	0.06	<0.001*
5-14 years	557	770,126,283	0.07	128	131,095,742	0.1	0.001*
15-24 years	3507	799,755,615	0.44	722	136,038,105	0.53	<0.001*
25-34 years	12,609	803,054,375	1.57	2,803	117,033,859	2.4	<0.001*
35-44 years	36,418	803,916,304	4.53	8,512	127,369,780	6.68	<0.001*
45-54 years	104,524	788,609,639	13.25	24,238	138,965,498	17.44	<0.001*
55-64 years	218,999	639,136,856	34.26	52,248	127,286,485	41.05	<0.001*
65-74 years	313,948	417,988,177	75.11	78,825	92,469,123	85.24	<0.001*
75-84 years	370,957	244,183,949	151.92	96,239	54,320,144	177.17	<0.001*
85+ years	275,680	98,324,522	280.38	73,606	21,189,280	347.37	<0.001*
Gender							
Male	689,811	2,815,055,490	24.5	170,343	502,292,400	33.91	<0.001*
Female	647,546	2,924,420,159	22.14	167,024	504,579,252	33.1	<0.001*
Race							
American Indian or Alaska Native	9,552	62,750,775	15.22	10,463	25,610,416	40.85	<0.001*
Asian or Pacific Islander	44,702	359,973,366	12.42	1,761	11,940,089	14.75	<0.001*
Black or African American	257,207	830,766,851	30.96	38,619	88,267,994	43.75	<0.001*
White	1,025,896	4,485,984,657	22.87	286,524	881,053,153	32.52	<0.001*

## Discussion

A retrospective original research study was conducted to analyze disparities in DM mortality over 22 years. This study revealed that mortality was consistently higher in rural areas throughout the study period. Over the last 22 years, the crude mortality in both urban and rural areas has shown an increasing trend. However, rural areas exhibited notably higher mortality, particularly in the 85+ age group, males, and Black or African American populations. A significant disparity was observed in the mortality rates of American Indians between urban and rural areas.

Given these findings, understanding the disparities in diabetes-related mortality between urban and rural areas is crucial for addressing the rising global burden of diabetes. Studies have consistently shown that rural populations face greater mortality risks from diabetes than their urban counterparts, which necessitates an investigation into the underlying causes and targeted interventions to bridge this gap [5,6].

Examining these disparities highlights inequities in healthcare access, quality, and outcomes between different regions [12]. Multiple factors contribute to these gaps. Disease-related complications are more prevalent in rural areas, often due to delayed diagnoses and inadequate disease management [13]. Area-specific challenges also play a role - rural regions generally have reduced access to advanced healthcare services, fewer medical professionals, and limited resources for diabetes education and management [7]. On the other hand, urban areas might see cases where patients present at more advanced stages of the disease due to socioeconomic barriers, further complicating timely healthcare access.

Studies like these show higher mortality rates in rural areas, underscoring the disproportionate burden of diabetes in these communities. This disparity is likely influenced by various factors, including limited healthcare infrastructure, lower socioeconomic status, and reduced access to preventive care [2]. These findings emphasize the urgent need for tailored interventions aimed at improving diabetes management and reducing mortality rates among rural populations.

When comparing the results of this study to existing literature, several similarities and distinctions were observed. The findings of this study suggest that rural areas experience significantly elevated diabetes-related mortality rates across all age groups, with older populations facing the most pronounced disparity (Figure 1). This is consistent with the study by Saydah et al., who found that rural populations encounter higher diabetes-related mortality rates due to challenges such as inadequate access to healthcare services and fewer medical resources in these settings [14]. However, this study also highlights a particularly high mortality rate in younger age groups (e.g., 25-34 years) in rural areas, a result that contrasts with studies such as Ali et al., which noted greater mortality in younger urban populations, likely due to lifestyle factors such as stress and sedentary behavior [15]. These differences may be attributed to the delayed access to healthcare and suboptimal management of diabetes in rural populations, resulting in more severe complications.

Limitations

This study has some important limitations that should be considered. First, the data analyzed in this research do not include the most recent years, specifically 2021 to 2023, which may affect the understanding of more recent trends in diabetes-related mortality. Additionally, the disease was not broken down into its subtypes, such as type 1 and type 2 diabetes, which could have provided more nuanced insights. Another limitation is that the specific causes of death related to diabetes were not studied, as the CDC WONDER database used in this analysis does not list such detailed information. This also prevented the authors from examining socioeconomic factors and healthcare access, which could be crucial in understanding the mortality disparities between urban and rural areas.

## Conclusions

This study reveals significant disparities in diabetes-related mortality between urban and rural populations, with rural areas facing considerably higher mortality rates, particularly among older adults, males, and Black or African American individuals. The findings emphasize the ongoing challenges in healthcare access and management that disproportionately affect rural communities, leading to worse outcomes in diabetes care. Further research is needed to uncover the root causes of these disparities, including the potential roles of socioeconomic status, access to healthcare services, and the management of diabetes in these regions. Policymakers should take these findings into account when designing targeted interventions aimed at lowering mortality rates in rural areas. Improving healthcare infrastructure, enhancing early diagnosis and diabetes management, and creating community-based health initiatives tailored to the specific needs of rural populations could be vital steps in reducing these disparities and improving health outcomes.
